# Soil-Transmitted Helminth Infections among Antenatal Women in Primary Care Settings in Southern India: Prevalence, Associated Factors and Effect of Anti-Helminthic Treatment

**DOI:** 10.3390/tropicalmed8010048

**Published:** 2023-01-07

**Authors:** Revathi Ulaganeethi, Ganesh Kumar Saya, Nonika Rajkumari, Swetha S. Kumar, Kalaiselvan Ganapathy, Gowri Dorairajan

**Affiliations:** 1 Department of Preventive and Social Medicine, Jawaharlal Institute of Postgraduate Medical Education and Research, Puducherry 605006, India; 2 Department of Microbiology, Jawaharlal Institute of Postgraduate Medical Education and Research, Puducherry 605006, India; 3 Department of Preventive and Social Medicine, Sri Manakula Vinayagar Medical College and Hospital, Puducherry 605602, India; 4 Department of Obstetrics and Gynecology, Jawaharlal Institute of Postgraduate Medical Education and Research, Puducherry 605006, India

**Keywords:** STH infections, pregnancy, cure rate, community-based, prevalence

## Abstract

Community-based studies from India on prevalence of soil-transmitted helminth (STH) infections have reported estimates as high as 50% in children. However, prevalence estimates during pregnancy in India are lacking. We aimed to describe the burden, associated factors of STH and cure rate after deworming in primary care settings. Pregnant women were recruited from four urban and five rural centers in Puducherry, South India, from December 2019 to April 2022. One stool sample was collected from each participant before deworming and one repeat sample was collected from STH positive woman after three weeks of deworming. The samples were processed with saline; iodine wet mount, and microscopic concentration techniques. Cure rate (CR) was assessed using Kato–Katz thick smear. Of 650 women included, 49 (7.5%, 95% CI 5.6–9.8) had one of the STH infections; the prevalence of *Ascaris lumbricoides,* hookworm and *Strongyloides* was 5.4%, 1.8% and 0.3%, respectively. The prevalence of any STH was higher among ages 26–30 years (9.1%), working women (8.3%), multigravida (8.3%), urban setting (8.3%), those who did not wash their hands before food (9%) and anemic women (8.9%), compared to their counterparts, but not statistically significant. The CR for hookworm was 100% and *Ascaris lumbricoides* was 88.6%. To conclude, the prevalence of STH was low among pregnant women compared to school aged children. Continued deworming activities along with improved sanitation could further reduce the burden.

## 1. Introduction

Protozoans and soil-transmitted helminths are the two main groups that cause intestinal parasitic infections (IPI) in humans and are a major public health concern in low-and middle income-countries (LMICs) [1]. Soil-transmitted helminths (STH) comprise *Ascaris lumbricoides, Necator americanus, Ancylostoma duodenale,* and *Trichuris trichura.* Globally more than 3.5 billion people were affected by one of the parasites, out of which 450 million were experiencing symptoms [2]. Annually, an estimated 44 million pregnancies are affected by soil transmitted helminths [3]. In India, the prevalence ranges from 12.5% to 66%, with a varying prevalence for individual parasites [4] and these infections are common where the sanitation and hygiene is poor [5].

As per World Health Organization, preschool children, school going children, adolescent and reproductive age group women are at greater risk of STH infection. Undernutrition (macronutrient and micronutrient) and repeated infections, including parasitic infections, lead to adverse consequences that can continue from one generation to the next [6]. Intestinal helminth infections adversely affect the food intake and digestion thereby causing iron depletion and anemia [7]. Additionally, hookworm infections in pregnancy results in chronic blood loss from the intestine and aggravates the development of iron-deficiency anemia. Increased physiological demands of iron during pregnancy combined with malnutrition and hookworm infection leads to severe iron deficiency anemia. This results in increased maternal mortality, inadequate weight gain, intra-uterine growth retardation, and a low birth weight [8,9,10].

In India, the prevalence of STH among children was reported as high as 50% in a systematic review published in 2017 [11]. A community-based study conducted during 2017–2018 in southern part of India reported an age-adjusted prevalence of 21% across all age groups [12]. In a tribal population in southern part of India, the prevalence was estimated to be 39% [13]. Hospital based studies in pregnant women in India have prevalence ranging from 8.3% to 12.4% [14,15]. To eliminate the morbidity of STH in preschool and school children by 2030, breaking the chain of transmission is essential. This could be attained by improving sanitation and hygiene practices with preventive strategies. Hence, WHO implemented biannual deworming with 400 mg of albendazole to all high-risk groups including pregnant women [16]. In India, all pregnant women receive deworming medication after the first trimester. Physiological and hormonal changes during pregnancy may alter the response to deworming. Development of resistance to albendazole may be a challenge in control efforts of STH infection. Hence it is essential to monitor the effect of deworming medication on controlling the parasitic infections.

The prevalence of intestinal parasitic infection and its associated factors were well studied in children, but not many studies have been done on pregnant women. Considering the importance and consequences of STH in pregnancy, routine community-based epidemiological studies are of prime importance in this high-risk group to know the baseline prevalence of infection. Hence, we undertook the study to estimate the prevalence of STH infections and its associated factors in pregnant women of Puducherry, India. We also reported cure rate (CR) and egg count reduction rate (ERR) after deworming medication.

## 2. Materials and Methods

### 2.1. Study Design and Setting

A community-based cross sectional study was conducted among pregnant women attending antenatal clinics in different primary health centers (PHCs) of Puducherry district during December 2019 to April 2022. Puducherry is a Union Territory in the southern part of India with a population of approximately 13 lakhs with 69% residing in urban areas and 31% in rural areas [17] Literacy rate was 91% and 80% in men and women respectively. Approximately 17% of the population was involved in agriculture related work. Approximately 75% of the employment was in services and industries (working as skilled and semi-skilled laborer’s) [18]. The estimated per capita income was around 210 thousand Indian rupees (~2546 US$) in the financial year 2021 [19]. Almost all households (99.9%) had access to improved drinking water source like piped water, protected dug well, tube well, bore well and community RO plant [20]. Majority of the houses were pucca houses (walls and roof made of permanent materials like concrete/galvanized sheets with cemented floor). 

**Figure 1:**
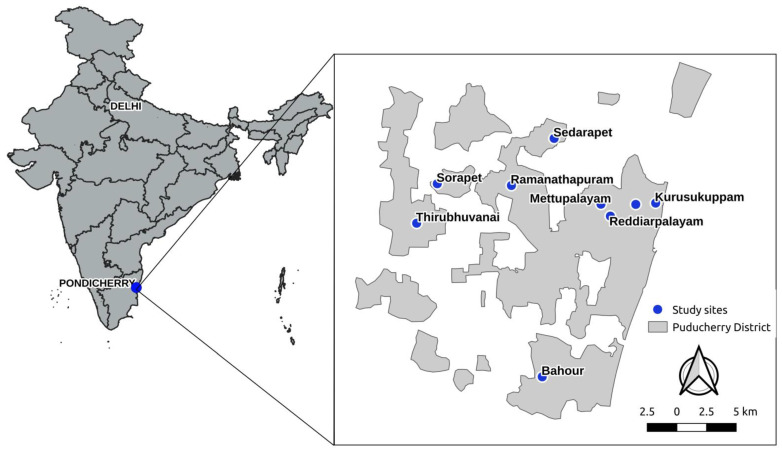
Map showing the location of the Primary health centers included in the study in Puducherry district, South India during December 2019–April 2022. Map was created using QGIS (Free open source software) and map data was obtained from Open Street Map.

Puducherry district has 27 primary health centers. Out of these, five urban and four rural were chosen by simple random sampling (Figure 1). In general, all pregnant women registered their pregnancy in PHCs in the first trimester. On registration, pregnant women received iron-folic acid and calcium tablets and tetanus toxoid injection. Deworming was given (a single dose of albendazole 400 mg) to all pregnant women during their second trimester of pregnancy without screening for a parasitic intestinal infection. All the supplements and deworming medications were provided free of cost. During the antenatal visits, hemoglobin was measured at the PHCs using a digital hemoglobinometer (Hbcheck, Biosense technologies Pvt.Ltd, Thane, Maharashtra, India).

### 2.2. Study Population

All pregnant women who were registered for antenatal care at selected PHCs (five urban and four rural) were considered as the source population. The women who visited the same during our sample collection period were considered our study population.

### 2.3. Inclusion and Exclusion Criteria

The pregnant women in any trimester of pregnancy before taking albendazole and willing to provide stool samples were included in the current study. Women who refused to provide stool samples were excluded.

### 2.4. Sample Size Estimation

Considering the prevalence of intestinal parasitic infection among pregnant women as 12.4% [15], absolute precision of 3, and design effect of 1.4, the calculated sample size was 649 for 95% confidence level. Open Epi version 3.01 was used for sample size calculation.

### 2.5. Methods

After obtaining the written informed consent, information regarding the women’s age, education, occupation, monthly household income, residence, gravida status, number of living children and behavioral characteristics were collected with the help of a questionnaire. Data on hemoglobin levels were extracted from individual case record of mothers and laboratory records maintained at PHCs. All the consecutive eligible pregnant women were enrolled in the study.

**Chart 1:**
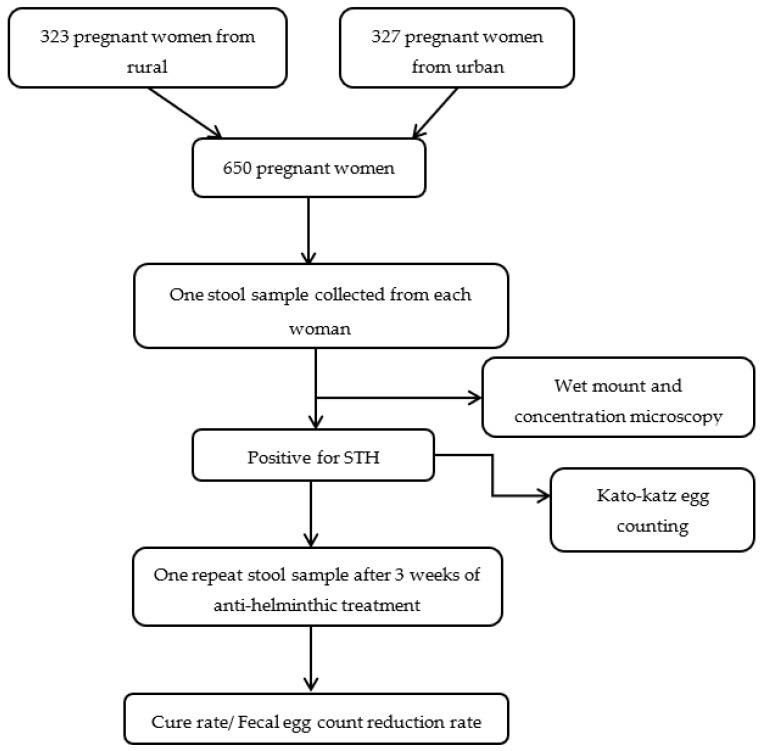
Flow diagram of study methodology.

A labeled screw-capped plastic container was given to the pregnant women for the stool sample collection, and they were instructed to collect stool sample without urine contamination. Stool samples were transported to the laboratory within four hours of collection. The samples were screened with saline, iodine wet mount, and 10% formol- ether sedimentation microscopic methods for the presence of helminths and other parasites. Additionally, Kato-Katz egg counting method was done for STH positive samples. We informed the results to all the women. Those women who were STH positive were referred to the medical officer of the PHC for management. Additionally, the line list of STH positive women was shared with the medical officers of PHC to ensure administration of deworming tablets (a single dose of Albendazole 400 mg) as treatment. After three weeks, a repeat sample was collected from STH positive women who received deworming medication. The repeat sample was screened with Kato-Katz to calculate the cure rate (CR) and egg reduction rate (ERR). The number of eggs were multiplied by 24 to derive the number of eggs per gram of feces [21]. The slides were examined under 10× and 40× magnifications. The cure rate was calculated by dividing the number of women who became STH negative after treatment by the total number of STH positive women. Fecal egg count reduction was defined as 1-(mean fecal egg count in the post-treatment/mean egg count in the pre-treatment)*100 [22]. The women infected with *Strongyloides* had been excluded for the calculation of CR and ERR Since albendazole was not the drug of choice for *strongyloides*. Flow Chart 1 described the methods in short.

### 2.6. Quality Assurance

The collected stool samples reached the laboratory within four of collection. Hence, the motility of the trophozoites was appreciated. All of the samples were read by a single investigator, and all the positive samples and 10% of the negative slides were cross-checked by the laboratory supervisor to confirm the results.

### 2.7. Statistical Analysis

The collected data was entered in the EpiData manager and analyzed using Stata version 14.0 [23]. The presence of STH was reported as proportions with 95% confidence intervals (CI). Age was converted into age categories and hemoglobin values were categorized into normal (≥11 gm/dL), mild (9–10.9 gm/dL), moderate (7–8.9 gm/dL) and severe (<7 gm/dL) anemia and reported as percentages. Association of socio-demographic, behavioral and obstetric factors with STH infection was assessed using Chi squared test and crude prevalence ratios with 95% CI were reported. A log binomial regression analysis was done to find the independent association of risk factors with STH infection A *p*-value of less than 0.05 was considered statistically significant. CR and ERR was calculated and expressed as percentages.

## 3. Results

### 3.1. Socio-Demographic and Obstetric Characteristics of Pregnant Women

Out of 650 pregnant women included, nearly half of them, 317 (48.8%), were aged less than 25 years and 372 (58%) had completed bachelor’s or master’s degrees. Approximately half of the women (N = 323, 49.7%) were from rural areas. Of all the women, 205 (31.5%) were working.

Of all the participants, 385 (59.2%) were primigravida. In total, 283 (43.8%) had mild anemia, and 51 (7.9%) had anemia of moderate grade and 2 (0.3%) had severe anemia. In total, 49 (7.5%) women reported taking deworming medication within one year before pregnancy. Table 1 describes the socio-demographic and obstetric characteristics of the pregnant women included in the study.

### 3.2. Behavioral Characteristics of Pregnant Women

A total of 598 (92%) and 539 (82.9%) women respectively reported washing their hands after using the toilet and before eating food, with soap and water. More than half, 411 (63.2%), reported walking barefoot outdoors sometimes or always, and 35 (5.4%) practiced open defecation. The majority of them, 571 (87.9%) were drinking tap water, and three fourths reported boiling of water before drinking (Table 2).

### 3.3. Prevalence of STH Infections

**Figure 2:**
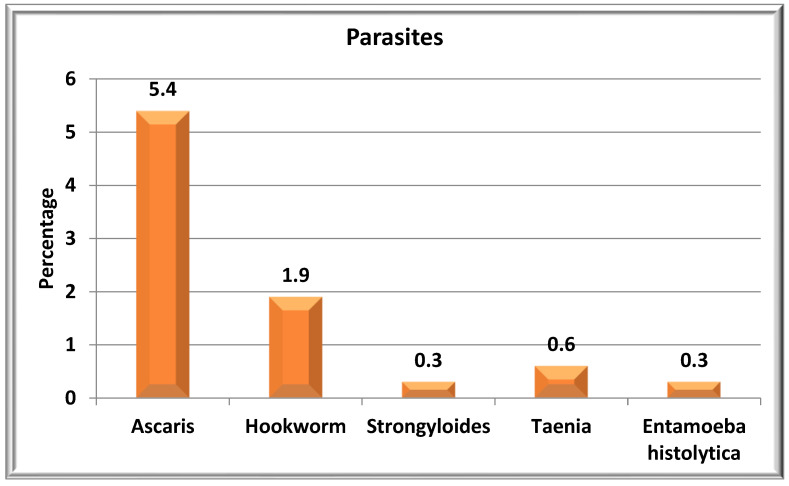
Prevalence of parasites among ante-natal women in primary care settings from Puducherry (N = 650).

Of 650 women, 49 (7.5%, 95% CI 5.6–9.8) were harboring any of the STH infection. The prevalence of *Ascaris lumbricoides* was the highest (5.4%, 35/650), followed by hookworm (1.9%, 12/650), and *Strongyloides* was seen in 2 (0.3%). Other parasite found were: *Taenia* (4/650) and *Entamoeba histolytica* (2/650) (Figure 2). The prevalence of any parasitic infection was 10.2%, (66/650, 95% CI: 7.9–12.7). One woman had a co-infection of *Ascaris lumbricoides* with hookworm species.

### 3.4. Factors Associated with STH Infections in Pregnancy

Prevalence of STH infections was 1.4 times higher in ages 26–30 years (9.1% vs. 6.3%) and 1.3 times higher in those more than 30 years (7.9% vs. 6.3%), when compared to 17–25 years. Nearly 1.4 times higher prevalence was observed in women residing in urban areas than rural (8.9% vs. 6.2%) The burden of STH was slightly higher in women who did not wash their hands before meals (9%), drank tap water (7.7%) and did not deworm in the previous year of pregnancy (7.7%) compared to women who washed their hands before food (7.2%), took purified water (6.3%) and who had dewormed in the previous year (6.1%). Though, these associations were not found to be statistically significant (Table 3). The prevalence of STH was higher in anemic women when compared to non-anemic women (5.8% in normal, 8.5% in mild, 9.8% in moderate and 50% in severe anemic) and an increasing trend was observed between the grading of anemia and STH prevalence (Chi squared for trend *p* value: 0.08).

### 3.5. Efficacy of Anti-Helminthic Treatment

Out of 47 women identified with STH infection other than *Strongyloides*, 43 had provided repeat stool samples after three weeks of deworming. The overall mean (SD) egg count in the pre and post treatment was 72.1 (35.2) and 3.3 (11.2) eggs per gram of stool. The overall cure rate was 90.7% (39/43, 95% CI 77.9–97.4). The mean (SD) egg count for *Ascaris lumbricoides* in the pre and post treatment was 72.1 (37.0) and 4.7 (13.0). For hookworm, it was 72 (30.7) in the pre-treatment and 0 count in the post treatment. The cure rate was 88.6% for *Ascaris lumbricoides* and 100% for hookworm. The egg reduction rate for *Ascaris* was 93.6%.

## 4. Discussion

The present study estimated the prevalence of STH infections among pregnant women and the effect of anti-helminthic treatment in primary care setting. The prevalence of STH was 7.5% and *Acaris lumbricoides* was predominant. Our study findings are similar to the prevalence estimates of 8.3% among pregnant women from a study conducted in the states of Rajasthan and Maharashtra from India [14]. Another hospital-based study from southern part of India reported the prevalence of 12.4%, which was slightly higher than the current study [15]. Another study done in Benin, Africa reported a prevalence of 13% and 9% in the first and second antenatal visits [24]. Two more studies in children had been published from the same setting reporting the prevalence of 34.6% in 2010 [4] and 6.4% in 2021 [25]. There was a major difference in the prevalence of helminth infection in our setting between 2010 and 2021. The difference might be due to the reason that in 2015, the Government of India launched a fixed day National Deworming day, wherein all children aged 1–19 years undergo deworming (single dose of Tab/Syrup) Albendazole 400 mg) to combat the infection [26]. In addition, Swachh Bharat Abhiyaan was also launched in 2014 to tackle the challenges related to water, sanitation, and hygiene [27]. These, with increased awareness, might reduce the existence of parasites in the study setting.

Other studies reported the burden of 51.5% in Ethiopia [28], 21.2% among refugees in Thailand [29], 38% in Gabon [30] and this was higher compared to the present study. Possible reasons for the high rate could be the differences in the method of diagnosis and poor living, environmental conditions and lack of awareness regarding the parasitic infections. 

The present study revealed that the prevalence was slightly higher in women practicing barefoot walking outdoors, and were more likely to be infected with STH. These observations were in line with the findings of a review [31] and a study conducted in Ethiopia [28]. Barefoot foot walking outdoors increases the probability of carrying contaminated soil to home, which could be the reason for higher STH infection. In addition to this, hookworm larva could directly penetrate the skin and cause infection. The current study observed a higher STH burden in women with anemia. Similar findings were observed in a study among pregnant women in southern India [15] and Myanmar [29]. In our study, the prevalence was slightly higher in urban settings than in rural. This was contradictory to the other studies [14,32]. Puducherry, being a union territory, had no marked difference in the living conditions, education level, and health access between rural and urban areas. In addition, barefoot walking, overcrowding and usage of public toilets especially in urban slums might be the reasons of higher prevalence in urban pockets. Overall, there has been an improvement in adapting good practices over last few decades and it is possible that the above factors may not play a major role in the STH transmission. Furthermore, false reporting of behavioral practices cannot be ruled out.

In our study, we achieved a cure rate of 88.6% for ascaris with albendazole and 100% for hook worm. A Cochrane review reported the cure rate of 93% for *ascaris* [33]. Another review among pregnant women found CR of 90% for both *ascaris* and hookworm [34]. A review done in children showed that the CR after 400 mg of albendazole for hookworm was 53.3% [35]. A study among Rwandan children showed an overall cure rate of 88.6% and an ERR of 75.4% by wet mount microscopy, and in our study the CR was 90.7% [22].The cure rates depend on the individual parasitic load and the presence of parasites in the environment, which could cause reinfection. In addition, the cure rate depends on the number of doses, quality of the drug, and dosage [36,37].

To our knowledge, this was the first community-based study on STH involving pregnant women, recruited from five rural and four urban primary care settings in southern part of India. A single observer screened all the samples for STH infection; hence inter-observer bias was avoided. The study also reported the effect of use of 400 mg of albendazole for treating STH during pregnancy in primary care setting. We had collected one stool sample from each participant, and the results relied on only microscopic techniques; thus, there is a possibility of under estimation of prevalence because of lower sensitivity. Additionally, previous studies have shown misclassification of artefacts as *Ascaris*, thus human errors in diagnosis of *Ascaris* eggs cannot be ruled out. Eggs of the two species of hookworm cannot be differentiated with microscopic techniques. Further molecular studies are advisable to confirm the diagnosis and species differentiation.

## 5. Conclusions

The prevalence of STH infections was less among pregnant women in our setting compared to other regions. Mass deworming programs with improvement in water, sanitation and hygiene (WASH) measures help to interrupt the transmission and reinfection in the community, thereby reducing the burden further. Periodic assessment of prevalence of STH in pregnancy is recommended and this will help in deciding the deworming strategies in the national programs.

## Figures and Tables

**Table 1:** Socio-demographic and obstetric characteristics of the pregnant women in primary care settings in Puducherry (N = 650).

Parameter	Number	Percentage
Age categories (Years)		
Up to 25	317	48.8
26–30	232	35.7
>30	101	15.5
Occupation		
Home maker	445	68.5
Working	205	31.5
Education		
No/Up to primary	17	2.7
Secondary to higher secondary	252	39.3
Bachelor/Master’s	372	58.0
Gravida		
Primi gravida	385	59.2
Multi gravida	265	40.8
No of living children		
None	402	61.9
One	223	34.3
Two or three	25	3.8
Residence		
Rural	323	49.7
Urban	327	50.3
Period of gestation		
first	282	43.4
second	347	53.4
third	21	3.2
Hemoglobin status (g/dl)		
Normal (≥11 gm/dl)	310	48.0
Mild (9–10.9 gm/dl)	283	43.8
Moderate (7–8.9 gm/dl)	51	7.9
Severe (<7 gm/dl)	2	0.3

**Table 2:** Behavioral characteristics of the pregnant women in primary care settings in Puducherry (N = 650).

Parameter	Number	Percentage
Handwashing after toilet use		
No/sometimes	52	8.0
Always	598	92.0
Handwashing before food		
No/sometimes	111	17.1
Always	539	82.9
Walking barefoot in outdoors		
No	239	36.8
sometime/always	411	63.2
Open defecation		
No	615	94.6
Yes	35	5.4
Drinking water		
Tap	571	87.9
Purified	79	12.2
Nature of water		
Boiled	150	23.1
Un boiled	500	76.9
Pet/Farm animal		
No	393	60.5
Yes	257	39.5

**Table 3:** Factors associated with STH infections among pregnant women recruited in primary care settings in Puducherry, South India. (N = 650).

Parameter	Total	Women with STH (*n* = 49)	Unadjusted PR	Adjusted PR	*p* Value
Age categories (Years)					
17–25	317	20 (6.3)	1	1	
26–30	232	21 (9.1)	1.4 (0.8–2.6)	1.3 (0.7–2.4)	0.4
>30	101	8 (7.9)	1.3 (0.6–2.8)	1.1 (0.5–2.5)	0.87
Education					
Up to primary school	17	0			
Secondary/Higher secondary	252	20 (7.9)	1.1 (0.7–1.8)		
Bachelor/Master’s degree	372	28 (7.5)	1		
Occupation					
Working	205	17 (8.3)	1.2 (0.7–2.0)		
Not working	445	32 (7.2)	1		
Gravida					
Primi	385	27 (7.0)	1		
Multi	243	22 (8.3)	1.2 (0.7–2.0)		
Number of living children					
None	402	27 (6.7)	1	1	
One	223	18 (8.1)	1.2 (0.7–2.3)	1.2 (0.7–2.1)	0.59
Two or three	25	4 (16.0)	2.4 (0.9–6.3)	1.3 (0.4–4.7)	0.7
Period of gestation					
First trimester	282	22 (7.8)	1.2 (0.7–2.1)	1.3 (0.7–2.3)	0.42
Second trimester	347	23 (6.6)	1	1	
Third trimester	21	4 (9.1)	2.9 (1.1–7.6)	3.1 (0.4–4.7)	0.02
Residence					
Rural	323	20 (6.2)	1	1	
Urban	327	29 (8.9)	1.4 (0.8–2.5)	1.5 (0.9–2.7)	0.14
Walking barefoot in outdoors					
Always/sometime	411	34 (8.3)	1.3 (0.8–2.4)		
No	239	15 (6.3)	1		
Handwashing before meals with soap					
No/some times	111	10 (9.0)	1.2 (0.6–2.4)		
Always	539	39 (7.2)	1		
Drinking water					
Tap	571	44 (7.7)	1.2 (0.5–3.0)		
Purified	79	5 (6.3)	1		
**Nature of water**					
Boiled	150	11 (7.3)	1		
Not boiled	500	38 (7.6)	1.03 (0.54–2.0)		
Handwashing after toilet use					
No/some times	52	2 (3.9)	0.5 (0.1–1.96)		
Always	598	47 (7.9)	1		
Raw/unwashed vegetable					
Sometimes	295	20 (6.8)	1		
Never	355	29 (8.2)	1.2 (0.7–2.1)		
Pet/Farm animals					
No	393	33 (8.4)	1.3 (0.8–2.4)		
Yes	257	16 (6.2)	1		
Hemoglobin (g/dl) *					
Normal (≥11)	310	18 (5.8)	1	1	
Mild anemia (9–10.9)	283	24 (8.5)	1.5 (0.8–2.6)	1.7 (1.0–3.2)	0.07
Moderate anemia (7–8.9)	51	5 (9.8)	1.7 (0.7–4.3)	2.0 (0.8–5.2)	0.16
Severe anemia (<7)	2	1 (50.0)	8.6 (2.0–37.0)	8.1 (1.3–50.5)	0.03
H/o deworming in the previous year					
Yes	49	3 (6.1)	1		
No	601	46 (7.7)	1.3 (0.4–3.9)		
Open defecation					
No	615	47 (7.6)	0.7 (0.2–3.0)		
Yes	35	2 (5.7)	1		

* Hemoglobin values were missing for four participants, PR-Prevalence ratio. *p* value: adjusted for others.

## Data Availability

The dataset used for this study are available with the corresponding author on a reasonable request.
